# Colonization with multidrug-resistant *Enterobacteriaceae* among infants: an observational study in southern Sri Lanka

**DOI:** 10.1186/s13756-021-00938-3

**Published:** 2021-04-30

**Authors:** Hannah R. Meredith, Sarath Kularatna, Kristin Nagaro, Ajith Nagahawatte, Champica Bodinayake, Ruvini Kurukulasooriya, Nishadhi Wijesingha, Lyndy B. Harden, Bhagya Piyasiri, Amr Hammouda, Brian M. Wiegmann, Bradly P. Nicholson, Maria Joyce, Christopher W. Woods, Arnoud H. M. Van Vliet, Siddhartha Thakur, L. Gayani Tillekeratne

**Affiliations:** 1 Department of Biomedical Engineering, Duke University, Durham, NC USA; 2 Department of Epidemiology, Johns Hopkins Bloomberg School of Public Health, Baltimore, MD USA; 3 Department of Obstetrics and Gynecology, Faculty of Medicine, University of Ruhuna, Galle, Sri Lanka; 4 Division of Infectious Diseases, Department of Medicine, Duke University School of Medicine, Durham, NC USA; 5 Department of Microbiology, Faculty of Medicine, University of Ruhuna, Galle, Sri Lanka; 6 Duke Global Health Institute, Duke University, Durham, NC USA; 7 Department of Medicine, Faculty of Medicine, University of Ruhuna, Galle, Sri Lanka; 8 Teaching Hospital Karapitiya, Galle, Sri Lanka; 9 Department of Entomology and Plant Pathology, NC State University, Raleigh, NC USA; 10 Institute of Medical Research, Durham, NC USA; 11 Durham Veterans Affairs Health System, Durham, NC USA; 12 Department of Pathology and Infectious Diseases, School of Veterinary Medicine, University of Surrey, Guildford, UK; 13 Department of Population Health and Pathobiology, NC State University, Raleigh, NC USA; 14 Comparative Medicine Institute, NC State University, Raleigh, NC USA

**Keywords:** ESBL, CRE, Multidrug-resistant *Enterobacteriaceae*, Sri Lanka, Intestinal colonization

## Abstract

**Background:**

The timing of and risk factors for intestinal colonization with multidrug-resistant *Enterobacteriaceae* (MDRE) are still poorly understood in areas with high MDRE carriage. We determined the prevalence, timing, and risk factors associated with MDRE intestinal colonization among infants in southern Sri Lanka.

**Methods:**

Women and their newborn children were enrolled within 48 h after delivery in southern Sri Lanka. Rectal swabs were collected from women and infants at enrollment and 4–6 weeks later. *Enterobacteriaceae* were isolated and identified as MDRE (positive for extended-spectrum β-lactamases or carbapenem resistant) using standard microbiologic procedures. We used exact methods (Fisher’s exact and Kruskal–Wallis tests) and multivariable logistic regression to identify sociodemographic and clinical features associated with MDRE intestinal colonization. Whole-genome sequencing was performed on selected MDRE isolates to identify phylogroups and antibiotic resistance-encoding genes were identified with NCBI’s AMRfinder tool.

**Results:**

Overall, 199 post-partum women and 199 infants were enrolled; 148/199 (74.4%) women and 151/199 (75.9%) infants were reassessed later in the community. Twenty-four/199 (12.1%) women and 3/199 (1.5%) infants displayed intestinal colonization with MDRE at enrollment, while 26/148 (17.6%) women and 24/151 (15.9%) infants displayed intestinal colonization with MDRE at the reassessment. While there were no risk factors associated with infant colonization at enrollment, multivariable analysis indicated that risk factors for infant colonization at reassessment included mother colonized at enrollment (aOR = 3.62) or reassessment (aOR = 4.44), delivery by Cesarean section (aOR = 2.91), and low birth weight (aOR = 5.39). Of the 20 MDRE isolates from infants that were sequenced, multilocus sequence typing revealed that 6/20 (30%) were clustered on the same branch as MDRE isolates found in the respective mothers. All sequenced isolates for mothers (47) and infants (20) had at least one ESBL-producing gene. Genes encoding fosfomycin resistance were found in 33/47 (70%) of mothers’ isolates and 16/20 (80%) of infants’ isolates and genes encoding resistance to colistin were found in one (2%) mother’s isolate.

**Conclusions:**

Our results suggest that a substantial proportion of infants undergo MDRE intestinal colonization within 6 weeks of birth, potentially due to postnatal rather than intranatal transmission.

**Supplementary Information:**

The online version contains supplementary material available at 10.1186/s13756-021-00938-3.

## Introduction

Infections due to multidrug-resistant *Enterobacteriaceae* (MDRE), such as extended-spectrum β-lactamase-producing and carbapenem-resistant *Enterobacteriaceae* (ESBL-E and CRE, respectively), are increasing in prevalence worldwide [1]. These infections are difficult to treat and associated with high morbidity and mortality [2]. The rise in ESBL-E and CRE infections has been more pronounced in regions such as the Middle East and South Asia, where a large proportion of otherwise healthy people (up to 70%) can asymptomatically carry ESBL-E or CRE in their gastrointestinal tracts [3, 4]. Since colonization is a precursor to infections such as bacteremia, pneumonia, and urinary tract infection, preventing MDRE colonization is an infection control priority.

In communities that have high baseline MDRE intestinal colonization prevalence, intranatal transmission (i.e., transmission at birth) or postnatal transmission (i.e., transmission through breastmilk, environment-to-person exposure, or person-to-person exposure after birth) may play an important role in MDRE intestinal colonization of infants. Limited available data have identified both intranatal risk factors, such as maternal colonization, and postnatal risk factors, such as water sources and pet exposure, for MDRE colonization [5–7]. Few of these published studies have been conducted in South Asia, where MDRE intestinal colonization prevalence is high (30–70% in South Asian countries as compared with < 15% in Europe) [3, 8]. In addition, conflicting conclusions exist as to whether exposures such as antibiotic use are risk factors for MDRE colonization [5, 9, 10].

In Sri Lanka, a south Asian country, the prevalence of MDRE intestinal colonization, age at which colonization occurs, and risk factors for colonization are unknown. We conducted an exploratory, prospective study in southern Sri Lanka to identify the prevalence, timing, and risk factors associated with MDRE intestinal colonization among infants in southern Sri Lanka.

## Methods

### Study design

The study was conducted in the post-partum wards of Mahamodara Teaching Hospital, the largest (400-bed) obstetric/gynecologic hospital in Southern Province, Sri Lanka, from August 2016 to January 2017.

### Study cohort and enrollment

Women (≥ 18 years old) who had presented in labor were approached within 48 h of delivery regarding study participation. Women who lived > 40 km from the hospital or who had active rectal bleeding were excluded. One rectal sample was collected from each woman and from each neonate using a FecalSwab (Copan Diagnostics) with Cary Blair media. If a patient was unable to tolerate rectal sampling, either a peri-rectal or fresh stool sample was collected using the swab. A standardized questionnaire was administered to collect sociodemographic information, clinical data, and exposure history. The medical record was reviewed during hospitalization to obtain information about the clinical course, such as receipt of antibiotics and clinical outcomes.

### Reassessment

Patients were visited at home 4–6 weeks after discharge to collect one rectal sample from each woman and infant using a method identical to that used at enrollment. A standardized questionnaire was administered to collect further details about exposures within the home environment.

### Microbiologic testing

Each swab was streaked on MacConkey agar with 2 μg/mL cefotaxime. Plates were incubated at 37 °C for 48 h and up to 3 morphologically different isolates per sample were selected for further characterization. Isolates were stored at − 70 °C until further analysis.

Species-level identification was performed using matrix-assisted laser desorption/ionization-time of flight mass spectrometry (MALDI-TOF, Bruker, Billerica, MA, USA). For organisms identified as *Enterobacteriaceae*, antibiotic susceptibilities and minimum inhibitory concentrations (MICs) were determined using MicroScan technology (Beckman Coulter, CA, USA), which automates organism identification and antimicrobial susceptibility testing results using microbroth dilution, according to Clinical and Laboratory Standards Institute guidelines [11]. Minimum inhibitory concentrations, as determined by microbroth dilution, were used to verify ESBL production. A decrease of ≥ 3 doubling dilutions in the MIC for either cefotaxime or ceftazidime tested in combination with 4 µg/mL clavulanic acid, versus its MIC when tested alone, was considered indicative of ESBL production. A modified Hodge test was performed on isolates with an intermediate or resistant susceptibility profile to ertapenem to detect the presence of carbapenemases. Phenotypic resistance to colistin and fosfomycin were confirmed by microbroth dilution, where a MIC > 4 μg/mL was considered indicative of colistin resistance and MIC > 128 μg/mL was considered indicative of fosfomycin resistance.

### Genome library preparation and sequence assembly

Whole genome sequencing of *Escherichia coli* isolates was conducted as previously described [12]. Briefly, the *E. coli* isolates (n = 76) were cultured overnight at 37 °C in Luria–Bertani media. Genomic DNA was extracted using a MasterPure Gram Positive DNA Purification Kit (Lucigen). DNA concentrations were quantitated using a Qubit 3.0 Fluorometer for double-stranded-DNA high-sensitivity assay kit (Thermo Fisher Scientific). The DNA was normalized and Illumina Nextera XT was used to prepare libraries: indexed libraries were generated using 12 cycles of PCR and each sample was indexed with a unique barcode. PCR cleanup and fragment size exclusion were done with an Ampure XP bead cleanup protocol to capture the desired band sizes for the DNA library (from ~ 250 bp to 1.0 kb). The fragmented DNA library was manually normalized and 24 libraries were pooled equally based on molar concentration and sequenced on the Illumina MiSeq v2 500 (2*250 bp paired-end reads) platform (MiSeq reagent kit, version 3). A PhiX control was spiked into each run as a quality control (QC). QC metrics monitored included clusters passing filter, base call quality scores (Q-Scores), cluster density, and estimated yields for each library and the PhiX control. FastQ files were generated and transferred to the NCBI SRA repository.

### Genome assembly and genomic analyses

FastQ files were obtained from the NCBI SRA repository using fastq-dump included in the SRA toolkit (https://www.ncbi.nlm.nih.gov/sra/docs/toolkitsoft/) (see SRA accession numbers in Additional File 1: Table S5). Genome sequences were assembled using Shovill version 1.0.4 (https://github.com/tseemann/shovill) using the SPAdes assembler with a coverage cut-off of 2 × and minimum contig size of 200 nt [13]. Assembly metrics were obtained using Quast version 4.6 [14]. The genome sequences were further characterized by determining the multilocus sequence type (ST) using MLST version 2.17.6 (https://github.com/tseemann/mlst) and the Warwick 7-gene scheme [15]. Serotypes were predicted using Abricate version 0.9.8 (https://github.com/tseemann/abricate) and the SerotypeFinder database [16]. The phylogroup of each isolate was determined using the ClermonTyping program version 1.4.1 [17]. Prediction of antimicrobial resistance was done using the Abricate program with the NCBI Bacterial Antimicrobial Resistance Reference Gene Database and the Resfinder database, and confirmed using AMRfinder version v3.1.1b [18, 19]. Phylogenetic trees were based on core genome single-nucleotide polymorphisms as identified using ParSNP version 1.2 using settings described previously [12, 20]. The final genomic sequences were uploaded to the designated NCBI Bioproject (PRJNA293225).

### Statistical analysis

Clinical data were entered into a Microsoft Access (Version 1808) database. To identify demographic features associated with MDRE intestinal colonization, Fisher’s exact and Kruskal–Wallis analyses were conducted for categorical and continuous data, respectively. Multivariable logistic regression analysis was also conducted to identify risk factors associated with MDRE intestinal colonization in infants and in mothers. Any risk factors associated with intestinal colonization with a *p* value < 0.05 on bivariable analysis were included in the multivariable model. Variables were checked for collinearity prior to inclusion in the model. As antibiotic use in mothers and delivery mode were collinear, antibiotic use in mothers was dropped from the multi-variable model for mothers. To create a more parsimonious model, risk factors were excluded in a step-wise manner until all *p* values were < 0.05. Each excluded variable was then added sequentially to ensure that it was not significant in the final model. Statistical analyses were conducted in STATA (Version 15.0).

### Ethical considerations

Ethical approval for this study was obtained from the Faculty of Medicine, University of Ruhuna, Ethical Review Committee (Sri Lanka) and the Duke University Institutional Review Board (USA).

## Results

### Study cohort

A total of 200 women and 202 infants (two sets of twins) were enrolled; however, samples from one woman and three infants were compromised in the storage process, thus leaving 199 women and 199 infants from enrollment for analysis. The median age for women was 29.0 years (IQR 25–33 years) and 95/199 (47.7%) of infants were female (Table 1, Additional File 1: Table S1). Most (85.4%) women were housewives or unemployed, and the majority (87.4%) had completed at least grade 10 education. The majority of women (158/199) reported a monthly household income ranging from 15,000 to 45,000 Rs (approximately 100–310 USD; Sri Lanka’s household average monthly income per capita was 115 USD in 2016) [21]. Reassessments were completed for 149/199 (75.4%) women and 151/199 (75.9%) infants; however, the sample from one woman was compromised during the storage process, thus leaving 148 women from reassessment for analysis. The median time between enrollment and the reassessment was 5.9 weeks (IQR 4.9–7.0 weeks) after hospital discharge.

**Table 1:** Sociodemographic and clinical features of infants at enrollment and reassessment

	Infant at admission				Infant at follow-up			
	All (n = 199)	MDRE + (n = 3)	MDRE– (n = 196)	*p* value	All (n = 151)	MDRE + (n = 24)	MDRE– (n = 127)	*p* value
General details								
Mother’s age (year)	29 (25–33)	32 (20–34)	29 (25–33)	0.749	29 (26–33)	30 (25–34)	29 (26–33)	0.129
Infant’s gender (female)	95 (47.7)	2 (66.7)	93 (47.5)	0.614	71 (47.0)	10 (41.7)	61 (48.0)	0.365
Low birth weight (< 2.5 kg)	19 (9.6)	0 (0)	19 (9.69)	1.000	16 (10.6)	6 (25)	10 (7.9)	0.023
Breastfed	198 (99.5)	3 (100)	195 (99.5)	0.985	151 (100)	24 (100)	127 (100)	1.000
Average monthly household income^a^	0.124				0.657			
< 15,000 Rs	10 (5.0)	0 (0)	10 (5.1)		9 (6.0)	0 (0)	9 (7.1)	
15,001–30,000 Rs	77 (38.7)	1 (33.3)	76 (38.8)		58 (38.4)	11 (45.8)	47 (37.0)	
30,001–45,000 Rs	82 (41.2)	0 (0)	82 (41.8)		62 (41.1)	10 (41.7)	52 (41.0)	
> 45,000 Rs	30 (15.1)	2 (66.7)	28 (14.3)		22 (14.6)	3 (12.5)	19 (15.0)	
Mother’s hospital-associated risk factors (within 6 months prior to delivery)								
Hospitalization	37 (18.6)	0 (0)	37 (18.9)	0.538	26 (17.2)	2 (8.33)	24 (18.9)	0.169
History of infection	9 (4.5)	0 (0)	9 (4.6)	0.870	9 (6.0)	1 (4.2)	8 (6.3)	0.566
Antibiotic intake	6 (3.0)	1 (33.3)	5 (2.6)	0.088	6 (4.0)	1 (4.2)	5 (3.9)	0.653
Household-associated risk factors (within 6 months prior to delivery)								
Number of adults at home	2 (1–3)	3 (1–4)	2 (1–3)	0.95	2 (1–3)	2 (1.5–3)	2 (1–3)	0.266
Number of children at home	1 (1–2)	1 (1–1)	1 (1–2)	0.64	1 (1–2)	1 (0–2)	1 (1–2)	0.214
Complications during pregnancy								
Any complications	22 (11.1)	0 (0)	22 (11.2)	0.702	20 (13.3)	5 (20.8)	15 (11.8)	0.189
Premature rupture of membranes	5 (2.51)	0 (0)	5 (2.6)	0.926	5 (3.3)	2 (8.3)	3 (2.4)	0.179
Gestational diabetes	6 (3.02)	0 (0)	6 (3.1)	0.912	6 (4.0)	2 (8.3)	4 (3.2)	0.243
Labor induced	13 (6.5)	0 (0)	13 (6.6)	1.000	11 (7.3)	1 (4.2)	10 (7.9)	1.000
Mode of delivery				0.559				0.021
C-section	64 (32.2)	0 (0)	64 (32.7)		51 (33.6)	13 (54.2)	38 (29.9)	
Vaginal	134 (67.3)	3 (100)	131 (66.8)		100 (66.2)	11 (45.8)	89 (70.1)	
Difficulties with delivery				0.592				0.133
No	62 (31.2)	0 (0)	62 (31.6)		49 (32.5)	13 (54.2)	36 (28.4)	
Episiotomy	131 (65.8)	3 (100)	128 (65.3)		97 (64.2)	11 (45.8)	86 (67.7)	
Hospitalization/discharge details								
NICU admission					3 (2.0)	1 (4.2)	2 (1.6)	0.407
Antibiotic started in hospital					3 (2.0)	1 (4.2)	2 (1.6)	0.407
Hospital stay—Infant (days)					1 (1–2)	2 (1–3)	1 (1–2)	0.060
Infant reassessment								
Admitted to hospital again					7 (4.6)	1 (4.2)	6 (4.7)	0.692
In-patient antibiotic use					4 (2.7)	0 (0)	4 (3.2)	1.000
Out-patient antibiotic use					21 (13.9)	6 (25)	15 (11.81)	0.087
Housing details								
Refrigerator					102 (67.6)	15 (62.5)	87 (68.5)	0.361
Running water					140 (92.7)	22 (91.7)	118 (92.9)	0.549
Toilet type								0.199
Outdoor squat toilet—private					116 (76.8)	23 (95.8)	93 (73.2)	
Outdoor squat toilet—public					32 (21.2)	1 (4.2)	31 (24.4)	
Private toilet/commode					2 (1.3)	0 (0)	2 (1.6)	
Pets at home					83 (55.0)	15 (62.5)	68 (53.5)	0.653
Household water treatment methods							0.204	
Boiled					67 (44.4)	9 (37.5)	58 (45.7)	
Filtered					21 (13.9)	5 (20.8)	16 (12.6)	
None					68 (45.0)	9 (37.5)	59 (46.5)	
Colonization								
Mother at enrollment	24 (12.1)	2 (66.7)	22 (11.2)	0.054	19 (12.6)	7 (29.2)	12 (9.5)	0.026
Mother at reassessment					26 (17.2)	9 (37.5)	17 (13.4)	0.012
Infant at enrollment					3 (2.0)	2 (8.3)	1 (0.8)	0.051

Risk factors associated with intestinal colonization with multidrug resistant *Enterobacteriaceae* (MDRE) on bivariable analysis are shown

Values are reported as frequency (%) or median (interquartile range). See Additional File 1: Table S1 for equivalent information regarding mothers

^a^< 15,000 Rs ≤ 89 USD; 15,001–30,000 Rs = 89–177 USD; 30,001–45,000 Rs = 177–266; > 45,000 Rs ≥ 266 USD

There were complications in 21/199 (10.6%) pregnancies: 4/199 (2.0%) had pre-mature rupture of membranes, 6/199 (3.0%) had gestational diabetes, and 13/199 (6.5%) had labor induced. Sixty-two/199 (31.2%) women underwent Caesarean section (C-section) and received prophylactic antibiotics prior to the procedure. In addition, 1/199 (0.5%) mother received antibiotics for treatment of infection during hospitalization. The median gestation period was 38.0 weeks (IQR 38.0–39.0 weeks). Nineteen/199 (9.6%) infants had low birth weight (< 2.5 kg). Most infants were breastfed at the time of enrollment (198/199, 99.5%). Antibiotics (including amoxicillin, a third-generation cephalosporin, penicillin, an aminoglycoside, and penicillin) were administered to 3/199 (1.5%) infants during hospitalization.

### MDRE intestinal colonization prevalence

Of the total cohort, rectal swabs from 40/199 (20.1%) mothers and 16/199 (8.0%) infants at enrollment and 41/148 (27.7%) mothers and 46/151 (30.5%) infants at reassessment resulted in growth of colonies on MacConkey with cefotaxime media. Of the isolates that grew on selective media, 3/40 (7.5%) of samples derived from mothers and 3/16 (18.8%) of samples derived from infants at enrollment and 4/41 (9.8%) of mothers’ and 12/46 (26.1%) of infants’ samples at the reassessment could not be re-cultured from storage, likely due to decreased viability associated with a freeze–thaw cycle, and were considered not to be MDRE for the purposes of the primary analysis. A sensitivity analysis in which these samples were evaluated as MDRE was conducted and showed no significant changes to the risk factors later discussed (Additional File 1: Table S2). Upon further analysis of the remaining isolates that grew on selective media, isolates from 12/40 (30%) of mothers at enrollment, 7/16 (43.8%) infants at enrollment, 10/41 (24.4%) of mothers at reassessment, and 10/46 (21.7%) of infants at reassessment were identified by MicroScan and MALDI-TOF as non-ESBL and non-CRE producing bacteria and thus categorized as non-MDRE. Only patients for whom isolates could be fully characterized as being MDRE were identified as being colonized with MDRE in the subsequent analyses.

**Fig. 1:**
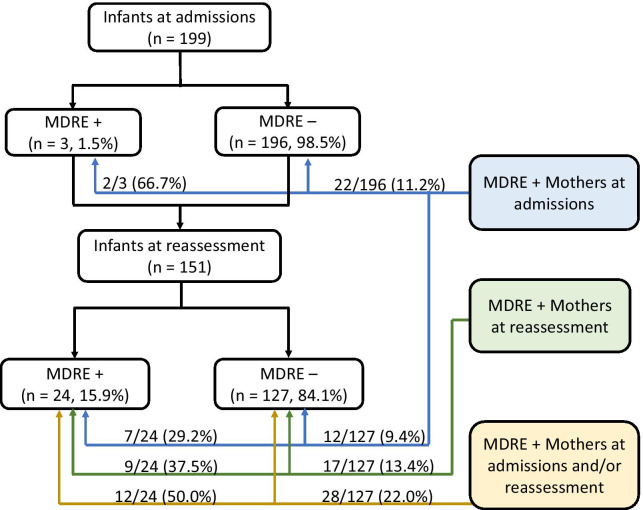
Flow chart of mothers and infants colonized with multidrug resistant *Enterobacteriaceae* (MDRE) at enrollment and reassessment. Counts inside the boxes represent the total number of infants for that category. Counts (proportion) on the lines represent the infants in that category who had MDRE + mothers at admissions (blue), reassessment (green), or admissions and/or reassessment (yellow)

Overall, 24/199 (12.1%) women and 3/199 (1.5%) infants had MDRE detected at enrollment, while 26/148 (17.6%) women and 24/151 (15.9%) infants had MDRE detected at reassessment (Fig. 1, Table 2). Antibiotic susceptibility testing results of MDRE isolates are documented in Additional File 1: Table S3. At enrollment and reassessment, respectively, 2/3 (66.7%) and 9/24 (37.5%) of MDRE-colonized infants also had MDRE-colonized mothers. Of the MDRE-colonized women at enrollment, 22/24 (91.7%) were colonized with ESBL-producing *E. coli* and 2/24 (8.3%) were colonized with ESBL-producing *Klebsiella pneumoniae*. Of the MDRE-colonized women at reassessment, all (26, 100%) were colonized with ESBL-producing *E. coli*. All MDRE-colonized infants at enrollment (3, 100%) had ESBL-producing *E. coli.* Of the MDRE-colonized infants at reassessment, 17/24 (70.8%) had ESBL-producing *E. coli*, 4/24 (16.7%) had ESBL-producing *K. pneumoniae*, and 4/24 (16.7%) had carbapenem-resistant *K. pneumoniae.* One infant at reassessment had both an ESBL-producing and carbapenem-resistant *K. pneumoniae* isolate. The modified Hodge test was negative in all CRE isolates.

**Table 2:** Prevalence of multidrug-resistant *Enterobacteriaceae* (MDRE) intestinal colonization in mothers and infants

	Mother (%)	Infant (%)
Enrollment (n_mother_ = 199, n_infant_ = 199)	24 (12.1)	3 (1.5)
ESBL *E. coli*	22 (91.7)	3 (100.0)
ESBL *K. pneumoniae*	2 (8.3)	0 (0)
Reassessment (n_mother_ = 148, n_infant_ = 151)	26 (17.6)	24 (15.9)
ESBL *E. coli*	26 (100.0)	17 (70.8)
ESBL *K. pneumoniae*	0 (0)	4 (16.7)
CRE *K. pneumoniae*	0 (0)	4 (16.7)
Overall (n_mother_ = 347, n_infant_ = 350)	50 (14.4)	27 (7.7)
ESBL *E. coli*	48 (96.0)	20 (71.4)
ESBL *K. pneumoniae*	2 (4.0)	4 (14.8)
CRE *K. pneumoniae*	0 (0)	4 (14.8)

Values are reported as frequency (proportion) of mothers or infants who were colonized by at least one isolate from a given category

*E. coli: Escherichia coli*;* K. pneumoniae: Klebsiella pneumoniae*; ESBL: extended spectrum β-lactamase; CRE: carbapenem resistant *Enterobacteriaceae*

### Risk factors for MDRE colonization

On unadjusted analysis, there were no significant risk factors associated with mothers’ or infants’ intestinal colonization with MDRE at enrollment. However, mothers were more likely to have intestinal colonization with MDRE at reassessment if they had more children at home (*p* = 0.001) (Additional File 1: Table S1). Infants were more likely to be intestinally colonized with MDRE at reassessment if they had been delivered by C-section (*p* = 0.021), had low birth weight (*p* = 0.023), or if the mother had intestinal colonization with MDRE at enrollment or reassessment (*p* = 0.026 and *p* = 0.012, respectively). Multivariable analysis indicated that mother’s colonization at enrollment (aOR = 3.62, 95% confidence interval (CI) = 1.04–12.57), mother’s colonization at reassessment (aOR = 4.44, CI = 1.38–14.30), delivery by C-section (aOR = 2.91, CI = 1.00–8.47), and low birth weight (aOR = 5.39, CI = 1.43–20.27) were positively associated with the infant’s intestinal colonization (Table 3). Complications during pregnancy or delivery, admission to the NICU, antibiotic use, source of nutrition, and water and animal exposures at home were not significantly associated with MDRE colonization.

**Table 3:** Multivariable analysis of risk factors associated with intestinal colonization with multidrug-resistant *Enterobacteriaceae* (MDRE) was conducted for infants at reassessment

Risk factor	Adjusted odds ratio (95% CI)
Mother colonized at enrollment	3.62 (1.04–12.57)
Mother colonized at reassessment	4.44 (1.38–14.30)
Infant colonized at enrollment	14.2 (0.82–245.22)
C-section delivery	2.91 (1.00–8.47)
Low birth weight	5.39 (1.43–20.27)

### Molecular analysis

Given funding restrictions, only *E. coli* isolates were selected for whole-genome sequencing. Of the *E. coli* isolates from mothers and infants, 47/54 (87.0%) from mothers and 20/22 (90.9%) from infants were selected for analysis; the remainder of *E. coli* isolates (4 in mothers and 2 in infants) were omitted due to poor DNA quality or were duplicates (3 in mothers). Analysis using the NCBI AMRfinder and Resfinder databases resulted in the identification of 61 genes that conferred resistance to antibiotics, as detailed in Additional File 1: Table S4. The five most common antibiotic resistance-encoding genes found in both mothers and infants were *bla*EC, *bla*CTX-M-15, gyrA-S83L, *mph*(A), and *bla*TEM-1. The median number of resistance genes reported for an isolate was 11 (IQR 7–16) and all isolates sequenced for mothers and infants had at least one ESBL-producing gene. Additionally, in the 47 isolates from mothers, genes encoding resistance to other classes of antibiotics were common: 38 (80.9%) to quinolones, 33 (70.2%) to fosfomycins, 28 (59.6%) to aminoglycosides or trimethoprim, 27 (57.5%) to sulfonamides, and 25 (53.2%) to macrolides (Table 4). Of note, one of the mother’s isolates carried the *mcr1.1* gene that encodes resistance to colistin, which was further confirmed by phenotypic screening. Of the 20 isolates from infants, 16 (80.0%) had genes encoding resistance to fosfomycin or trimethoprim and 15 (75.0%) had genes encoding resistance to quinolones or aminoglycosides, and 14 (70.0%) had genes encoding resistance to macrolides.

**Table 4:** Summary of antimicrobial resistance present in *E. coli* isolates found in mothers and infants

	Isolates from infants			Isolates from mothers		
	Overall n = 20	Admissions n = 2	Reassessment n = 18	Overall n = 47	Admissions n = 21	Reassessment n = 26
Aminoglycosides	15 (75.0)	1 (50.0)	14 (83.3)	28 (59.6)	10 (47.6)	18 (69.2)
Antiseptics	10 (50.0)	1 (50.0)	9 (50.0)	20 (42.6)	10 (47.6)	10 (38.5)
β-lactams	20 (100.0)	2 (100.0)	18 (100.0)	47 (100.0)	21 (100.0)	26 (100.0)
Chloramphenicol	4 (20.0)	1 (50.0)	3 (16.8)	14 (29.8)	6 (28.6)	8 (30.8)
Colistin	0 (0)	0 (0)	0 (0)	1 (2.1)	0 (0)	1 (3.9)
Fosfomycin	16 (80.0)	2 (100.0)	14 (77.8)	33 (70.2)	13 (61.9)	20 (76.9)
Macrolides	14 (70.0)	1 (50.0)	13 (72.2)	25 (53.2)	11 (52.4)	14 (53.9)
Quinolones	15 (75.0)	2 (100.0)	13 (72.2)	38 (80.9)	18 (85.7)	20 (76.9)
Sulfonamides	11 (55.0)	1 (50.0)	10 (55.6)	27 (57.5)	12 (57.1)	15 (57.7)
Tetracyclines	11 (55.0)	1 (50.0)	10 (55.6)	19 (40.4)	9 (42.9)	10 (38.5)
Trimethoprim	16 (80.0)	1 (50.0)	15 (83.3)	28 (59.6)	13 (61.9)	15 (57.7)

Values reported as frequency (proportion) of *E. coli* isolates detected carrying the relevant resistance genes. Note that more than one isolate was cultured from some participants and that four isolates from mothers and two isolates from infants were omitted due to poor DNA quality. See Additional File 1: Table S4 for specific genes

**Fig. 2:**
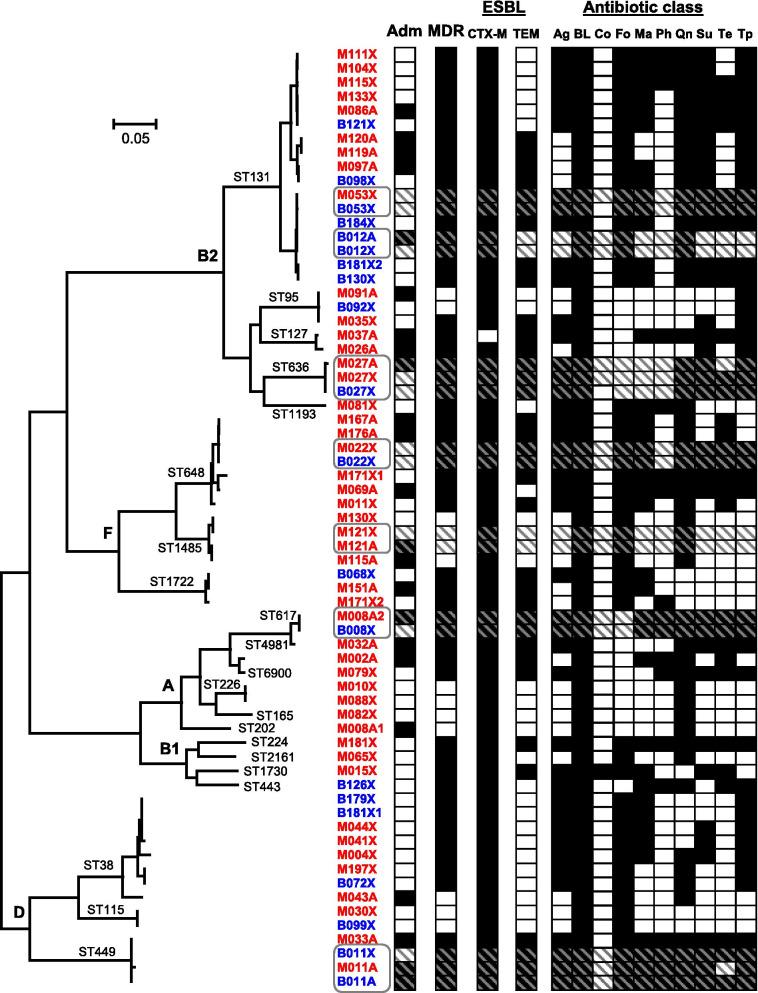
Phylogenetic relationship and antimicrobial resistance prediction of genome sequences from *Escherichia coli* isolated during admission and reassessment. The isolates were clustered based on core genome SNPs identified using ParSNP [12, 20], with the phylogroups B2, F, A, B1 and D indicated at the appropriate branching points in the tree. Sample names are provided, with red labeled text highlighting samples obtained from mothers, dark blue highlighting samples obtained from infants, with the column “Adm” showing *E. coli* isolated from samples obtained at initial admission (black) or reassessment (white). Antimicrobial genotypes were predicted using AMRfinder, and grouped per class of antibiotics, with multidrug resistance (MDR, predicted resistance to ≥ 3 classes of antibiotics). All isolates were predicted to contain extended spectrum β-lactamases (ESBL), with the next two columns indicating the presence (black) or absence (white) of *bla*CTX-M and *bla*TEM genes. The final ten bars show the presence (black) and absence (white) of individual classes of antibiotic resistances (Ag, aminoglycoside; BL, β-lactam; Co, colistin; Fo, fosfomycin; Ma, macrolide; Ph, phenicol; Qn, quinolone; Su, sulfonamide; Te, tetracycline; Tp, trimethoprim). Full data with individual resistance genes and mutations can be found in Additional File 2: Table S8. Grey ellipses and stripes indicate the mothers and/or infants that are clonally related (nomenclature: M = mother, B = infant, A = admissions, X = reassessment, number = household ID)

The sequenced isolates fell into five phylogroups (A, B1, B2, D, and F), (Fig. 2, Additional File 1: Tables S5–S7). Phylogroup B2 was the most common overall (34% and 50% of sequenced isolates from mothers and infants, respectively), followed by phylogroups F, D, A, and B1. Multilocus sequence typing (MLST) revealed that isolates from phylogroup B2 were mostly ST131 (56.3% and 80.0% of phylogroup B2 found in mothers and infants, respectively), phylogroup D were mostly ST38 (56.3% and 50.0% of phylogroup D in mothers and infants, respectively), and phylogroup F were mostly ST648 (50.0% and 50.0% of phylogroup F in mothers and infants, respectively). Of the 20 sequenced isolates from infants, 6 (30%) were clustered on the same branch as the mother’s isolates, 10 (50.0%) isolates were from infants whose mothers were not detected to be colonized with multidrug-resistant *E. coli*, and 4 (20.0%) were clustered on branches different from the mother’s isolate. The mother-infant pairs with isolates clustered on the same branch were distributed evenly across all phylogroups (except for phylogroup B1, which had none), suggesting there was no single strain that was more likely to be transmitted from mother to infant. Of the infants colonized at enrollment, 2/3 (66.7%) had a similar isolate detected at reassessment, suggesting that early colonization may not be transient. Infants colonized at reassessment (whose mothers were not colonized) were distributed across the phylogroups and had varied resistance profiles, further suggesting that there was not a common source for or strain of MDREs.

## Discussion

Understanding how and when intestinal colonization occurs is important for designing control strategies that reduce MDRE colonization prevalence. Of interest is the Middle East and South Asia, where MDRE colonization prevalence is higher than in other parts of the world but not widely studied. We present the first report on prevalence of MDRE colonization in Sri Lanka, where a high (12–17.6%) proportion of women were colonized with MDRE. While only 1.5% of infants were colonized with MDRE at birth, this number increased to 15.9% by 6 weeks of age. Our study is hypothesis generating, and suggests that postnatal MDRE colonization via contact transmission in the community, including from mother’s kissing, skin-to-skin contact, or breastmilk, may be more common than intranatal colonization for infants.

The prevalence of intestinal MDRE colonization was high in women in this study, relative to Western settings (12–17.6% vs 1–6%, respectively) [22–24]. The reason for the higher prevalence in our study is likely multi-factorial, but different practices in antibiotic use and access may be important. One study showed that the majority (80%) of outpatients with likely viral respiratory illnesses in Sri Lanka were prescribed antibiotics [25]. Similarly, a survey of pharmacy students in Sri Lanka reported that 76% of them had used antibiotics in the last year [26]. This overuse of antibiotics is driven by multiple factors, including physicians’ perception that patients desire antibiotics, clinicians’ inability to discern viral from bacterial infections based on symptoms, and patients’ ability to obtain over-the-counter antibiotics [26, 27]. Our intestinal colonization prevalence in women is similar to figures reported previously from some studies in low- and middle-income countries (India 15% ESBL-E colonization in 2007–2009, Tanzania 15% ESBL-E colonization in 2013, and Madagascar 18.5% ESBL-E colonization in 2014) [3, 6, 9, 28].

The observation of colonization of infants within 6 weeks after birth in this study corroborates limited data from other studies in areas of high MDRE colonization prevalence [10, 29]. A study set in India showed that infant colonization with ESBL-E increased from 14.3% after birth to 41.5% in 60 days [10]. A study set in Spain reported that 19.6% of infants with ESBL-E-colonized mothers were colonized with ESBL-E at birth and 22.2% were colonized after 3 months [29]. The latter number decreased to 11.1% after 1 year. Overall, these prevalence rates are high and reflective of the level of endemicity in a given community. Relative to these studies, we reported a lower initial colonization prevalence in infants, possibly reflective of a lower colonization prevalence in mothers; however, we saw a similar rapid colonization in the weeks following birth.

Colonization of mothers as a significant factor in infant colonization corroborates data from other studies [5, 29]. Infants delivered vaginally are exposed to their mothers’ intestinal microbiota, including MDRE that may be present [30]. Infants delivered by C-section had a higher risk of being colonized with MDRE at reassessment. Since women who delivered by C-section received a prophylactic antibiotic, the risk could be due to the selective pressure of the antibiotic and/or the exposure to a different microbiome. As infants delivered by C-section are colonized by different bacteria than those delivered vaginally, it could be easier for MDRE to become established in their microbiome [31, 32]. Additionally, studies have shown that intestinal bacteria can be present in breastmilk, posing another way by which an infant may be exposed to a mother’s microbiome [32, 33].

Genotyping revealed that a quarter of colonized infants were colonized with a strain found in the same cluster as their mother’s isolate and more than half of colonized infants at reassessment did not have colonized mothers. This finding supports data from other studies that found little-to-no similarity in ESBL isolates between mothers and their infants [9, 29]. All of the sequenced MDRE isolates carried CTX-M genes and about one-third harbored OXA genes, conferring resistance to a wide range of β-lactam antibiotics. This finding is consistent with observations in other South and Southeast Asian countries that have reported a high prevalence of CTX-M and OXA enzymes [34, 35]. Interestingly, we did not detect OXA-23, reportedly the most prevalent oxacillinase today. In addition to β-lactam resistance, genotyping and phenotypic screening detected isolates with resistance to a wide variety of other antibiotics. A large proportion of isolates were resistant to quinolones, supporting previous findings that ESBL production is often associated with quinolone resistance [36]. Isolates were also detected with resistance to colistin and fosfomycin, which are considered antibiotics of last resort in most settings [37]. To our knowledge, this is the first time colistin and fosfomycin resistance have been reported in Sri Lanka, and this finding is further evidence of the worrisome rise in antibiotic resistance.

Molecular typing of *E. coli* isolates revealed that phylogroup B2 was the most common among our isolates, with ST131 being the predominant sequence type within this group. Our findings are consistent with data from around the world which suggest that phylogroup B2 and ST131 are prevalent among ESBL-producing *E. coli* globally [38]. *E. coli* ST131 in particular has spread throughout the world, commonly carrying the ESBL-encoding gene CTX-M-15 and encoding multidrug resistance [39]. In our study, all of our ST131 isolates carried this antibiotic resistance gene.

Some limitations in our study must be noted. Single rectal swabs may have limited the ability to detect MDRE isolates and may have led to low estimates of relatedness between individuals [40]. Consequently, the ability to accurately characterize transmission routes and common sources of MDRE (either hospital or community) was limited. Variations in single swab detection sensitivity could explain the discrepancy observed with some mothers being colonized only at enrollment or reassessment (not both) and could lead to an underestimation of MDRE intestinal colonization. Repeated swabs, using an enriched broth detection method, or additional time points would increase the chances of capturing a MDRE isolate and shed light on the degree to which MDRE colonization is transient [41]. Another source of MDRE prevalence underestimation may have been that some isolates initially grew on the MacConkey and cefotaxime selective media but could not be re-cultured. This loss of isolates could be due to decreased viability of organisms following a freeze–thaw cycle. However, a sensitivity analysis assessing for risk factors associated with MDRE colonization showed no changes from the primary analysis when these lost isolates were considered as MDRE. Ultimately, the loss of isolates during the storage process or during the freeze–thaw cycle would only have under-estimated the number of women and infants reported to be colonized with MDRE. We were only able to perform reassessments on 75% of subjects, and we did not follow patients beyond 6 weeks after enrollment. While the modified Hodge test that we performed was recommended by the CLSI for phenotypic confirmation of carbapenemase production, other tests, such as the Carba NP test or modified Carbapenemase Inactivation Method (mCIM), have improved specificity and sensitivity and could have provided a more sensitive method for detecting carbapenemases. Finally, the study’s limited funding precluded whole genome sequencing of the *K. pneumoniae* isolates; thus, the antibiotic resistance genes, phylogroups, and relatedness between mother and infant of these MDRE remain to be characterized. In addition, while our study has highlighted a high prevalence of MDRE intestinal colonization, the association of MDRE colonization with subsequent infection needs to be further characterized.

## Conclusion

In conclusion, MDRE intestinal colonization was established within 6 weeks of birth among infants in Sri Lanka. Our results suggest that infants were likely colonized during the first few weeks after birth, possibly due to colonization from an intranatal or nosocomial exposure, contact transmission in the community, or breastmilk. Identifying how and when MDRE colonization occurs will be critical for preventing such colonization, which may be a precursor to MDRE infections that are challenging to treat.

## Supplementary Information

**Additional file 1: Table S1.** Description of mothers at enrollment and reassessment in addition to bivariable analysis of risk factors associated with intestinal colonization with multidrug resistant Enterobacteriaceae (MDRE). **Table S2.** Sensitivity analysis for definition of isolates that were not re-culturable. **Table S3.** Summary of antibiotic resistance by phenotypic susceptibility testing of isolates found in mothers and infants. **Table S4.** Summary of antibiotic resistance-encoding genes present in isolates found in mothers and infants. **Table S5.** Information on *E. coli* isolates characterized using whole genome sequencing. **Table S6**. Mothers’ isolates categorized by phylogroup and Multilocus Sequence Typing (MLST). **Table S7**. Infants’ isolates categorized by phylogroup and Multilocus Sequence Typing (MLST).

**Additional file 2: Table S8.** Antimicrobial resistance prediction of *E. coli* genomes included in this study, as determined by AMRfinder.

## Data Availability

Data from the surveys and microbiological testing are available upon request. The final genomic sequences were uploaded to the designated NCBI Bioproject (PRJNA293225).

## Abbreviations

MDRE: Multidrug-resistant *Enterobacteriaceae*
ESBL-E: Extended-Spectrum β-lactamase-producing *Enterobacteriaceae*
CRE: Carbapenem-Resistant *Enterobacteriaceae*
