# Supplementary material for: Colonization with multidrug-resistant Enterobacteriaceae among infants: an observational study in southern Sri Lanka
Source: Antimicrob Resist Infect Control. 2021 Apr 30;10:72. doi: 10.1186/s13756-021-00938-3 (PMC8086278; doi:10.1186/s13756-021-00938-3)
Supplement: Supplementary file 1 — Additional file 1: Table S1. Description of mothers at enrollment and reassessment in addition to bivariable analysis of risk factors associated with intestinal colonization with multidrug resistant Enterobacteriaceae (MDRE). Table S2. Sensitivity analysis for definition of isolates that were not re-culturable. Table S3. Summary of antibiotic resistance by phenotypic susceptibility testing of isolates found in mothers and infants. Table S4. Summary of antibiotic resistance-encoding genes present in isolates found in mothers and infants. Table S5. Information on E. coli isolates characterized using whole genome sequencing. Table S6. Mothers’ isolates categorized by phylogroup and Multilocus Sequence Typing (MLST). Table S7. Infants’ isolates categorized by phylogroup and Multilocus Sequence Typing (MLST). [file 13756_2021_938_MOESM1_ESM.docx]

Colonization with multidrug-resistant *Enterobacteriaceae* among infants: an observational study in southern Sri Lanka – Additional File 1

Hannah R. Meredith^1,2^, Sarath Kularatna^3^, Kristin Nagaro^4^, Ajith Nagahawatte ^5,6^, Champica Bodinayake^,6,7^, Ruvini Kurukulasooriya^5^, Nishadhi Wijesingha^5^, Lyndy B. Harden^8^, Bhagya Piyasiri^9^, Amr Hammouda^12^, Brian Wiegmann^10^, Bradly P. Nicholson^11^, Maria Joyce^4,12^, Christopher W. Woods^4,6,12^, Arnoud H. M. van Vliet^13^, Siddhartha Thakur^14,15^, L. Gayani Tillekeratne^4,6,7,12^

^1^ Department of Biomedical Engineering, Duke University, Durham, NC

^2^ Department of Epidemiology, Johns Hopkins Bloomberg School of Public Health, Baltimore, MD

^3^ Department of Obstetrics & Gynecology, Faculty of Medicine, University of Ruhuna, Galle, Sri Lanka

^4^ Division of Infectious Diseases, Department of Medicine, Duke University School of Medicine, Durham, NC

^5^ Department of Microbiology, Faculty of Medicine, University of Ruhuna, Galle, Sri Lanka

^6^ Duke Global Health Institute, Duke University, Durham, NC

^7^ Department of Medicine, Faculty of Medicine, University of Ruhuna, Galle, Sri Lanka

^8^ College of Veterinary Medicine, NC State University, Raleigh, NC

^9^ Teaching Hospital Karapitiya, Galle, Sri Lanka

^10^ Department of Entomology and Plant Pathology, NC State University, Raleigh, NC

^11^ Institute of Medical Research, Durham, NC

^12^ Durham Veterans Affairs Health System, Durham, NC

^13^ Department of Pathology and Infectious Diseases, School of Veterinary Medicine, University of Surrey, Guildford, UK

^14^ Department of Population Health & Pathobiology, NC State University, Raleigh, NC

^15^ Comparative Medicine Institute, NC State University, Raleigh, NC

**Table S1.** Sociodemographic and clinical features of mothers at enrollment and reassessment. Risk factors associated with intestinal colonization with multidrug resistant Enterobacteriaceae (MDRE) on bivariable analysis are shown. Values are reported as frequency (%) or median (interquartile range).

| **Mothers at enrollment** | **All**  (n = 199) | **MDRE +**  (n = 24) | **MDRE -**  (n = 175) | **p-value** |
| --- | --- | --- | --- | --- |
| Age (yr) | 29 (25-33) | 28 (25.5-33) | 29 (25-33) | 0.236 |
| Height (cm) | 154 (150-157) | 153.5 (149-157) | 154 (150-157) | 0.655 |
| Weight (kg) | 62 (57-70) | 64 (58-73) | 62 (56-69.5) | 0.845 |
| BMI (kg/m^2^) | 26.8 (24.5-28.9) | 27.5 (25.3-28.8) | 26.7 (24.1 - 29.2) | 0.722 |
| # of adults at home | 2 (1-3) | 3 (1.5-4) | 2 (1-3) | 0.605 |
| # of children at home | 1 (1-2) | 1 (0-1) | 1 (1-2) | 0.951 |
| **Marital status** |  |  |  | 0.879 |
| Married | 198 (99.5) | 24 (100) | 174 (99.43) |  |
| Divorced | 0 (0) | 0 (0) | 0 (0) |  |
| Single | 1 (0.5) | 0 (0) | 1 (0.57) |  |
| **Education** |  |  |  | 0.712 |
| <O Level | 25 (12.56) | 4 (16.67) | 21 (12) |  |
| O Level | 101 (50.75) | 10 (41.67) | 91 (52) |  |
| A Level | 63 (31.66) | 9 (37.5) | 54 (30.86) |  |
| >A Level | 10 (5.03) | 1 (4.17) | 9 (5.14) |  |
| **Average monthly household income** |  |  |  | 0.412 |
| < 15000 Rs | 10 (5.03) | 1 (4.17) | 9 (5.14) |  |
| 15001-30000 Rs | 75 (37.69) | 10 (41.67) | 66 (37.14) |  |
| 30001-45000 Rs | 83 (41.71) | 7 (29.17) | 76 (43.43) |  |
| > 45000 Rs | 31 (15.58) | 6 (25) | 25 (14.29) |  |
| **Food consumption patterns** |  |  |  |  |
| Chicken, meat, fish | 198 (99.5) | 24 (100) | 174 (99.43) | 0.879 |
| Dairy | 198 (99.5) | 24 (100) | 174 (99.43) | 0.879 |
| Greens | 193 (96.98) | 24 (100) | 169 (96.57) | 0.458 |
| **Hospital associated risk factors (past 6 months)** |  |  |  |  |
| Hospitalization | 37 (18.59) | 2 (8.33) | 35 (20) | 0.133 |
| History of infection | 9 (4.52) | 2 (8.33) | 7 (4) | 0.297 |
| **Bath water sources** |  |  |  |  |
| Tap | 78 (39.2) | 13 (54.17) | 65 (37.14) | 0.230 |
| Wells in general | 121 (60.8) | 11 (45.83) | 110 (63.86) | 0.226 |
| **Drinking water sources** |  |  |  |  |
| Tap | 77 (38.89) | 12 (50) | 65 (37.14) | 0.356 |
| Wells in general | 122 (61.31) | 12 (50) | 110 (62.86) | 0.161 |
| **Drinking water treatment methods** |  |  |  |  |
| Boiled | 47 (23.63) | 5 (20.83) | 42 (24) | 0.480 |
| Filtered | 40 (20.1) | 5 (20.83) | 35 (20) | 0.553 |
| None | 120 (60.3) | 14 (58.33) | 106 (60.57) | 0.500 |
| **Medical history in past 6 months** |  |  |  |  |
| Diabetes mellitus | 6 (3.02) | 1 (4.17) | 5 (2.86) | 0.542 |
| Hypertension | 1 (0.5) | 0 (0) | 1 (0.57) | 0.879 |
| Chronic respiratory disease | 3 (1.5) | 0 (0) | 3 (1.71) | 0.679 |
| Family member admitted to hospital | 9 (4.52) | 3 (12.5) | 6 (3.43) | 0.079 |
| Antibiotic intake in past 6 months | 6 (3.02) | 2 (8.33) | 4 (2.29) | 0.155 |
| **Complications during pregnancy** |  |  |  |  |
| Any complications | 21 (10.55) | 2 (8.33) | 19 (10.86) | 0.521 |
| Premature rupture of membranes | 4 (2.01) | 0 (0) | 4 (2.29) | 0.596 |
| Gestational diabetes | 6 (3.02) | 1 (4.17) | 5 (2.86) | 0.542 |
| Labor induced | 13 (6.53) | 1 (4.17) | 12 (6.86) | 1.00 |
| **Mode of delivery** |  |  |  | 0.215 |
| C-section | 62 (31.16) | 11 (45.83) | 51 (29.14) |  |
| Vaginal | 136 (68.34) | 13 (42.17) | 123 (70.29) |  |
| **Difficulties with delivery** |  |  |  | 0.139 |
| None | 60 (30.15) | 9 (37.5) | 51 (29.14) |  |
| Episiotomy | 133 (66.83) | 13 (54.17) | 120 (68.57) |  |
| Antibiotic therapy started in hospital | 63 (31.66) | 11 (45.83) | 52 (29.71) | 0.089 |
| Days in hospital before delivery | 1 (0-1) | 1 (0-1) | 1 (0-1) | 0.859 |
| **Mothers at reassessment** | **All**  (n = 148) | **MDRE +**  (n = 26) | **MDRE -**  (n = 122) | **p-value** |
| Age (yr) | 29 (26-33) | 29 (26-34) | 29 (26-34) | 0.811 |
| Height (cm) | 154 (150-157) | 155 (149-158) | 153.5 (150-157) | 0.237 |
| Weight (kg) | 63 (57-70) | 62 (57-70) | 63.25 (57-70) | 0.694 |
| BMI (kg/m^2^) | 27.1 (24.06 - 29.52) | 26.8 (23.12-29.52) | 27.06 (24.26-29.67) | 0.218 |
| # of adults at home | 2 (1-3) | 3 (1-3) | 2 (1-3) | 0.345 |
| # of children at home | 1 (0.5-2) | 1.5 (1-2) | 1 (0-2) | 0.001 |
| Hospitalization details |  |  |  |  |
| Mother colonized at enrollment | 19 (12.84) | 5 (19.23) | 14 (11.48) | 0.45 |
| Mother's hospital duration (days) | 2 (1-3) | 2 (1-4) | 2 (1-3) | 0.075 |
| Antibiotic therapy started in hospital | 50 (33.78) | 7 (26.92) | 43 (35.25) | 0.283 |
| Fever since discharge Mother | 15 (10.14) | 4 (15.38) | 11 (9.02) | 0.256 |
| Mother admitted to hospital again | 1 (0.68) | 1 (3.85) | 0 (0) | 0.176 |
| In-patient antibiotic use | 1 (0.68) | 1 (3.85) | 0 (0) | 0.176 |
| Out-patient antibiotic use | 23 (15.54) | 6 (23.08) | 17 (13.93) | 0.189 |
| **Housing details** |  |  |  |  |
| Refrigerator | 101 (68.24) | 14 (53.85) | 87 (71.31) | 0.068 |
| Running water | 138 (93.24) | 23 (88.46) | 115 (94.26) | 0.246 |
| **Toilet type** |  |  |  | 0.908 |
| private squat toilet outdoors | 113 (76.35) | 20 (76.92) | 93 (76.23) |  |
| public squat toilet outdoors | 32 (21.62) | 6 (23.08) | 26 (21.31) |  |
| private toilet/commode | 2 (1.35) | 0 (0) | 2 (1.64) |  |
| **Pets kept at the house**: |  |  |  |  |
| No pets | 69 (46.62) | 10 (38.46) | 59 (48.36) | 0.24 |
| Any pet | 82 (55.41) | 19 (73.08) | 63 (51.64) | 0.12 |

**Table S2.** Sensitivity analysis: multivariable analysis of risk factors associated with intestinal colonization with multidrug-resistant Enterobacteriaceae (MDRE). Potential MDRE samples that could not be re-cultured following a freeze-thaw cycle were regarded as non-MDRE in the primary analysis. This sensitivity analysis shows that risk factors associated with colonization are similar when the lost samples are considered to be MDRE.

|  | Uncultured samples = MDRE- | Uncultured samples = MDRE+ | |
| --- | --- | --- | --- |
|  | Adjusted Odds Ratio  (95% CI) | Adjusted Odds Ratio  (95% CI) |  |
| **Mother at admission** |  |  |  |
| History of infection | 1.5 (0.24-8.99) | 1.4 (0.23 – 8.23) |  |
| Number of children at home | 0.8 (0.47 – 1.31) | 0.7 (0.41 – 1.15) |  |
| Family member admitted to hospital | 3.6 (0.75 -17.21) | 3.2 (0.67 – 15.10) |  |
| **Mother at reassessment** |  |  |  |
| Mother colonized at enrollment | 1.9 (0.54 – 6.82) | 1.7 (0.50-5.65) |  |
| Number of children at home | 2.0 (1.17 – 3.38) | 2.1 (1.28 – 3.51) |  |
| Number of adults at home | 1.3 (0.88 – 1.88) | 1.2 (0.87-1.76) |  |
| Antibiotics taken during hospital stay | 0.7 (0.26 – 2.10) | 0.8 (0.31 – 2.18) |  |
| Mother hospital duration | 1.0 (0.98 – 1.11) | 1.0 (0.97 – 1.1) |  |
| **Infant at reassessment** |  |  |  |
| Mother colonized at enrollment | 3.6 (1.04 – 12.57) | 3.8 (1.33-10.80) |  |
| Mother colonized at reassessment | 4.4 (1.38 – 14.30) | 3.5 (1.37-9.00) |  |
| Infant colonized at enrollment | 14.2 (0.82– 245.22) | 1.2 (0.17-9.00) |  |
| C-section delivery | 2.9 (1.00 – 8.47) | 1.8 (0.75-4.17) |  |
| Low birth weight | 5.4 (1.43 – 20.27) | 3.6 (1.08-11.8) |  |

**Table S3.** Summary of antibiotic susceptibility testing results among MDRE isolates in mothers and infants. Values reported as frequency (proportion) of isolates that did not test “susceptible” to the given antibiotic (inclusive of “intermediate” and “resistant” results). Please note that some participants were colonized with more than one isolate.

|  | Isolates from infants | | | Isolates from mothers | | |
| --- | --- | --- | --- | --- | --- | --- |
|  | Overall  n = 31 | Admission  n = 3 | Reassessment  n = 28 | Overall  n = 56 | Admission n = 25 | Reassessment  n = 31 |
| Aminoglycoside | 14 (45.16) | 0 (0) | 14 (50) | 11 (19.64) | 3 (12) | 8 (25.81) |
| Amikacin | 0 (0) | 0 (0) | 0 (0) | 1 (1.79) | 0 (0) | 1 (3.23) |
| Gentamicin | 10 (32.26) | 0 (0) | 10 (35.71) | 6 (10.71) | 0 (0) | 6 (19.35) |
| Tobramycin | 14 (45.16) | 0 (0) | 14 (50) | 11 (19.64) | 3 (12) | 8 (25.81) |
| β-lactams | 31 (100) | 3 (100) | 28 (100) | 56 (100) | 25 (100) | 31 (100) |
| Ampicillin/Sulbactam | 24 (77.42) | 2 (66.67) | 22 (78.57) | 38 (67.86) | 16 (64) | 22 (70.97) |
| Ampicillin | 31 (100) | 3 (100) | 28 (100) | 56 (100) | 25 (100) | 31 (100) |
| Amoxicillin/Clavulanate | 16 (51.61) | 1 (33.33) | 15 (53.57) | 18 (32.14) | 12 (48) | 6 (19.35) |
| Aztreonam | 31 (100) | 3 (100) | 28 (100) | 56 (100) | 25 (100) | 31 (100) |
| Ceftriaxone | 31 (100) | 3 (100) | 28 (100) | 56 (100) | 25 (100) | 31 (100) |
| Ceftazidime | 31 (100) | 3 (100) | 28 (100) | 56 (100) | 25 (100) | 31 (100) |
| Cefotaxime | 31 (100) | 3 (100) | 28 (100) | 56 (100) | 25 (100) | 31 (100) |
| Cefoxitin | 5 (16.13) | 0 (0) | 5 (17.86) | 6 (10.71) | 4 (16) | 2 (6.45) |
| Cefazolin | 31 (100) | 3 (100) | 28 (100) | 56 (100) | 25 (100) | 31 (100) |
| Cefepime | 31 (100) | 3 (100) | 28 (100) | 56 (100) | 25 (100) | 31 (100) |
| Cefuroxime | 31 (100) | 3 (100) | 28 (100) | 56 (100) | 25 (100) | 31 (100) |
| Ertapenem | 4 (12.9) | 0 (0) | 4 (14.29) | 0 (0) | 0 (0) | 0 (0) |
| Imipenem | 4 (12.9) | 0 (0) | 4 (14.29) | 0 (0) | 0 (0) | 0 (0) |
| Meropenem | 4 (12.9) | 0 (0) | 4 (14.29) | 0 (0) | 0 (0) | 0 (0) |
| Piperacillin/Tazobactam | 31 (100) | 3 (100) | 28 (100) | 56 (100) | 25 (100) | 31 (100) |
| Piperacillin | 31 (100) | 3 (100) | 28 (100) | 56 (100) | 25 (100) | 31 (100) |
| Quinolone | 19 (61.29) | 1 (33.33) | 18 (64.29) | 27 (48.21) | 7 (28) | 20 (64.52) |
| Ciprofloxacin | 19 (61.29) | 1 (33.33) | 18 (64.29) | 27 (48.21) | 7 (28) | 20 (64.52) |
| Levofloxacin | 15 (48.39) | 1 (33.33) | 14 (50) | 26 (46.43) | 7 (28) | 19 (61.29) |
| Moxifloxacin | 15 (48.39) | 1 (33.33) | 14 (50) | 27 (48.21) | 7 (28) | 20 (64.52) |
| Sulfonamides | 22 (70.97) | 1 (33.33) | 21 (75) | 37 (66.07) | 14 (56) | 23 (74.19) |
| Trimethoprim/  Sulfamethozaxole | 22 (70.97) | 1 (33.33) | 21 (75) | 37 (66.07) | 14 (56) | 23 (74.19) |
| Tetracycline | 19 (61.29) | 2 (66.67) | 17 (60.71) | 27 (48.21) | 11 (44) | 16 (51.61 |
| Tetracycline | 19 (61.29) | 2 (66.67) | 17 (60.71) | 27 (48.21) | 11 (44) | 16 (51.61) |
| Tigecycline | 0 (0) | 0 (0) | 0 (0) | 0 (0) | 0 (0) | 0 (0) |

**Table S4.** Antibiotic resistance-encoding genes and their frequency in E. coli from mothers and infants colonized with MDRE. Data from samples collected at admission and at reassessment are aggregated in the table. Values are reported as frequency (proportion) of isolates.

|  | **NCBI** | | **ResFinder** | | | **Function** |
| --- | --- | --- | --- | --- | --- | --- |
|  | **Mother (%)**  **(n = 47)** | **Infant (%)**  **(n = 20)** | | **Mother (%)**  **(n = 47)** | **Infant (%)**  **(n = 20)** |  |
| **aac(3)-IIa** | 2 (4.3) | 0 (0) | | 2 (4.3) | 0 (0) | Confers resistance to aminoglycosides (Ag), [1] |
| **aac(3)-IId** | 1 (2.1) | 1 (5) | | 1 (2.1) | 1 (5) |  |
| **aadA1** | 7 (14.9) | 6 (30) | | -- | -- |  |
| **aadA2** | 2 (4.3) | 0 (0) | | 2 (4.3) | 0 (0) |  |
| **aadA5** | 11 (23.4) | 7 (35) | | 11 (23.4) | 7 (35) |  |
| **ant(3”)-Ia** | -- | -- | | 7 (14.9) | 6 (30) |  |
| **aph(3')-Ia** | 1 (2.1) | 0 (0) | | 1 (2.1) | 0 (0) |  |
| **aph(3'')-Ib** | 19 (40.4) | 10 (50) | | 19 (40.4) | 10 (50) |  |
| **aph(6)-Id** | 19 (40.4) | 10 (50) | | 19 (40.4) | 10 (50) |  |
| **aac(6')-Ib-cr** | 6 (12.8) | 2 (10) | | 7 (14) | 2 (10) | Confers resistance to aminoglycosides (Ag) and fluoroquinolones (Fo) [2] |
| **blaCTX-M** | 0 (0) | 1 (5) | | - | - | Confers resistance to β-lactams [3] |
| **blaCTX-M-14** | 5 (10.6) | 1 (5) | | 5 (10.6) | 1 (5) |  |
| **blaCTX-M-15** | 38 (80.9) | 15 (75) | | 38 (80.9) | 16 (80) |  |
| **blaCTX-M-27** | 2 (4.3) | 3 (15) | | 2 (4.3) | 3 (15) |  |
| **blaCTX-M-182** | 1 (2.1) | 0 (0) | | 1 (2.1) | 0 (0) |  |
| **blaTEM** | 1 (2.1) | 0 (0) | | - | - |  |
| **blaTEM-1** | 22 (46.8) | 11 (55) | | 24 (51.1) | 11 (55) |  |
| **blaTEM-40** | 2 (4.3) | 0 (0) | | - | - |  |
| **blaTEM-214** | - | - | | 1 (2.1) | 0 (0) |  |
| **blaOXA** | 7 (14.9) | 2 (10) | | - | - |  |
| **blaOXA-1** | 7 (14.9) | 4 (20) | | 14 (29.8) | 6 (30) |  |
| **blaDHA-1** | 1 (2.1) | 0 (0) | | 1 (2.1) | 0 (0) |  |
| **blaEC** | 46 (97.9) | 20 (100) | | - | - |  |
| **blaEC-5** | 1 (2.1) | 0 (0) | | - | - |  |
| **catA1** | 6 (12.8) | 2 (10) | | 6 (12.8) | 2 (10) | Confers resistance to chloramphenicol [4] |
| **catB3** | 9 (19.2) | 2 (10) | | 7 (14.9) | 2 (10) |  |
| **dfrA1** | 5 (10.6) | 4 (20) | | 4 (8.5) | 4 (20) | Confers resistance to trimethoprim [5] |
| **dfrA5** | 6 (12.8) | 2 (10) | | 6 (12.8) | 2 (10) |  |
| **dfrA7** | 2 (4.3) | 2 (10) | | 2 (4.3) | 2 (10) |  |
| **dfrA8** | 1 (2.1) | 0 (0) | | 1 (2.1) | 0 (0) |  |
| **dfrA12** | 2 (4.3) | 0 (0) | | 2 (4.3) | 0 (0) |  |
| **dfrA14** | 1 (2.1) | 1 (5) | | 1 (2.1) | 1 (5) |  |
| **dfrA17** | 11 (23.4) | 7 (35) | | 11 (23.4) | 7 (35) |  |
| **erm(B)** | 6 (12.8) | 2 (10) | | 6 (12.8) | 2 (10) | Confers resistance to macrolides [6] |
| **mph(A)** | 23 (48.9) | 14 (70) | | 23 (48.9) | 14 (70) |  |
| **cyaA_S352T** | 20 (42.6) | 8 (40) | | - | - |  |
| **fosA3** | 1 (2.1) | 0 (0) | | 1 (2.1) | 0 (0) | Confers resistance to fosfomycin [7,8] |
| **ptsl_V25I** | 9 (19.2) | 8 (40) | | - | - |  |
| **uhpT_E350Q** | 16 (34.0) | 11 (55) | | - | - |  |
| **mcr-1.1** | 1 (2.1) | 0 (0) | | 1 (2.1) | 0 (0) | Confers resistance to colistin [9] |
| **mdf(A)** | - | - | | 47 (100) | 20 (100) | Confers resistance to lipophilic compounds, chloramphenicol, erythromycin, fluroquinolones, aminoglycosides (Ag), neomycin, and kanamycin [10] |
| **qacE** | 1 (2.1) | 0 (0) | | - | - | Confers resistance to anti-septics [11,12] |
| **qacEdelta1** | 19 (40.4) | 10 (50) | | - | - |  |
| **gyrA_D87N** | 16 (34.0) | 6 (30) | | - | - | Confers resistance to quinolones [13,14] |
| **gyrA_D87Y** | 1 (2.1) | 0 (0) | | - | - |  |
| **gyrA_S83L** | 25 (53.2) | 14 (70) | | - | - |  |
| **parC_E84V** | 5 (10.6) | 1 (5) | | - | - |  |
| **parC_S57T** | 2 (4.3) | 2 (10) | | - | - |  |
| **parC_S80I** | 17 (36.2) | 6 (30) | | - | - |  |
| **parE_E460D** | 0 (0) | 2 (10) | | - | - |  |
| **parE_I529L** | 9 (19.2) | 6 (30) | | - | - |  |
| **parE_L416F** | 1 (2.1) | 1 (5) | | - | - |  |
| **qnrB4** | 1 (2.1) | 0 (0) | | 1 (2.1) | 0 (0) |  |
| **qnrS** | 1 (2.1) | 0 (0) | | -- | -- |  |
| **qnrS1** | 10 (21.3) | 0 (0) | | 10 (21.3) | 0 (0) |  |
| **sul1** | 19 (40.4) | 10 (50) | | 19 (40.4) | 10 (50) | Confers resistant to sulfonamides [15] |
| **sul2** | 16 (34.0) | 9 (45) | | 16 (34.0) | 9 (45) |  |
| **sul3** | 1 (2.1) | 0 (0) | | 1 (2.1) | 0 (0) |  |
| **tet(A)** | 7 (14.9) | 5 (25) | | 7 (14.9) | 5 (25) | Confers resistance to tetracycline [16] |
| **tet(B)** | 12 (25.5) | 6 (30) | | 12 (25.5) | 6 (30) |  |
| **tet(M)** | 1 (2.1) | 0 (0) | | 1 (2.1) | 0 (0) |  |

**Table S5**. Information on E. coli isolates characterized using whole genome sequencing. M = mother, B = infant, A = admissions, X = reassessment, three digit number = household ID, trailing number distinguishes isolate number when more than one is detected per participant. All sequences have been uploaded under Bioproject PRJNA293225.

| Tree order‡ | | Sample | Phylogroup | | MLST* | O-antigen† | H-antigen† | ESBL type | SRA accession number |
| --- | --- | --- | --- | --- | --- | --- | --- | --- | --- |
|  | |  |  | |  |  |  |  |  |
| 1 | | M111X | B2 | | 131 | O25 | H4 | CTX-M-15 | SRR8360148 |
| 2 | | M104X | B2 | | 131 | O25 | H4 | CTX-M-15, TEM-1, OXA-1 | ~ |
| 3 | | M115X | B2 | | 131 | O25 | H4 | CTX-M-15 | SRR9050355 |
| 4 | | M133X | B2 | | 131 | O25 | H4 | CTX-M-15, OXA-1 | ~ |
| 5 | | M086A | B2 | | 131 | O25 | H4 | CTX-M-15, TEM-1 | SRR8360117 |
| 6 | | B121X | B2 | | 131 | O25 | H4 | CTX-M-15, OXA-1 | SRR8360109 |
| 7 | | M120A | B2 | | 131 | O25 | H4 | CTX-M-15, TEM-1 | SRR8360116 |
| 8 | | M119A | B2 | | 131 | O25 | H4 | CTX-M-15, OXA-1 | ~ |
| 9 | | M097A | B2 | | 131 | O25 | H4 | CTX-M-182, TEM-1 | SRR8360119 |
| 10 | | B098X | B2 | | 131 | O25 | H4 | CTX-M-15, TEM-1, OXA-1 | SRR8984096 |
| 11 | | M053X | B2 | | 131 | O16 | H5 | CTX-M-15, TEM-1, OXA-1 | SRR8535168 |
| 12 | | B053X | B2 | | 131 | O16 | H5 | CTX-M-15 | SRR9050395 |
| 13 | | B184X | B2 | | 131 | O16 | H5 | CTX-M-15 | SRR9050394 |
| 14 | | B012A | B2 | | 131 | O16 | H5 | CTX-M-15, TEM-1, OXA-1 | ~ |
| 15 | | B012X | B2 | | 131 | O16 | H5 | CTX-M-15, TEM-1, OXA-1 | ~ |
| 16 | | B181X2 | B2 | | 131 | O16 | H5 | CTX-M-27, TEM-1 | SRR9050389 |
| 17 | | B130X | B2 | | 131 | O16 | H5 | CTX-M-15 | ~ |
| 18 | | M091A | B2 | | 95 | O50-O2 | H4 | CTX-M-15, OXA-1 | SRR8360146 |
| 19 | | B092X | B2 | | 95 | O50-O2 | H4 | CTX-M-15, TEM-1 | ~ |
| 20 | | M035X | B2 | | 95 | O50-O2 | H4 | CTX-M-15, TEM-1 | SRR8535144 |
| 21 | | M037A | B2 | | 127 | O6 | H31 | CTX-M-15, TEM-1 | SRR9050387 |
| 22 | | M026A | B2 | | 127 | noO | H31 | CTX-M-15 | SRR8360112 |
| 23 | | M027A | B2 | | 636* | noO | H7 | CTX-M-15, TEM-1, OXA-1 | SRR8535119 |
| 24 | | M027X | B2 | | 636 | O21 | H7 | CTX-M-15, TEM-1 | SRR8535119 |
| 25 | | B027X | B2 | | 636 | O21 | H7 | CTX-M-186 | SRR8360104 |
| 26 | | M081X | B2 | | 1193 | O75 | H5 | CTX-M-15 | SRR8360142 |
| 27 | | M167A | F | | 648 | O1 | H6 | CTX-M-15, TEM-1 | SRR8360150 |
| 28 | | M176A | F | | 648 | O1 | H6 | CTX-M-15 | ~ |
| 29 | | M022X | F | | 648 | O1 | H6 | CTX-M-15, TEM-1, OXA-1 | SRR8663255 |
| 30 | | B022X | F | | 648 | O1 | H6 | CTX-M-15, TEM-1, OXA-1 | SRR9050361 |
| 31 | | M171X1 | F | | 648 | O102 | H6 | CTX-M-15 | ~ |
| 32 | | M069A | F | | 648 | O1 | H6 | TEM-1 | SRR8535177 |
| 33 | | M011X | F | | 648 | O1 | H6 | CTX-M-15 | ~ |
| 34 | | M130X | F | | 1485 | O83 | H42 | CTX-M-15 | SRR8360227 |
| 35 | | M121X | F | | 1485 | O83 | H42 | CTX-M-15, OXA-1 | SRR8360263 |
| 36 | | M121A | F | | 1485 | O83 | H42 | CTX-M-15, OXA-1 | SRR8360139 |
| 37 | | M115A | F | | 1485 | O83 | H42 | CTX-M-27 | SRR8360151 |
| 38 | | B068X | F | | 1722 | O130 | H45 | CTX-M-14, TEM-1 | SRR9050337 |
| 39 | | M151A | F | | 1722 | O130 | H45 | CTX-M-15, TEM-1 | ~ |
| 40 | | M171X2 | F | | 1722 | O11 | H25 | CTX-M-15 | ~ |
| 41 | | M008A2 | A | | 617 | O89-O162 | H10 | CTX-M-15, TEM-1, OXA-1 | SRR8535116 |
| 42 | | B008X | A | | 617 | O89-O162 | H10 | CTX-M-15, TEM-1, OXA-1 | SRR8360138 |
| 43 | | M032A | A | | 4981 | O89-O162 | H9 | CTX-M-14, TEM-1 | SRR8535153 |
| 44 | | M002A | A | | N/A | O89-O162 | H9 | CTX-M-15 | SRR8360261 |
| 45 | | M079X | A | | 6900 | O89-O162 | H9 | CTX-M-15 | SRR8535163 |
| 46 | | M010X | A | | 226 | O8-O40 | H4 | CTX-M-14 | SRR8535139 |
| 47 | | M088X | A | | 226 | O8-O40 | H4 | CTX-M-15 | SRR8360111 |
| 48 | | M082X | A | | 165 | noO | H26 | CTX-M-15 | SRR8360110 |
| 49 | | M008A1 | A | | 202 | noO | noH | CTX-M-15, TEM-1 | ~ |
| 50 | | M181X | B1 | | 224 | O9 | H30 | CTX-M-27 | SRR8360267 |
| 51 | | M065X | B1 | | 2161 | O180 | H14 | CTX-M-15, TEM-40, OXA-1 | SRR8535160 |
| 52 | | M015X | B1 | | 1730 | noO | H10 | CTX-M-15, TEM-1, OXA-1 | SRR8535138 |
| 53 | | B126X | B1 | | 443 | noO | H21 | CTX-M-15, TEM-1 | SRR8360114 |
| 54 | | B179X | D | | 38 | O86 | H30 | CTX-M-27 | SRR8360258 |
| 55 | | B181X1 | D | | 38 | O86 | H30 | CTX-M-15, OXA-1 | SRR8360255 |
| 56 | | M044X | D | | 38 | O86 | H30 | CTX-M-15, TEM-1 | SRR8535151 |
| 57 | | M041X | D | | 38* | noO | H30 | CTX-M-15, TEM-1 | ~ |
| 58 | | M004X | D | | 38 | O86 | H18 | CTX-M-27, TEM-1 | SRR8360256 |
| 59 | | M197X | D | | 38 | noO | H30 | CTX-M-15, TEM-1, OXA-1 | SRR8360253 |
| 60 | | B072X | D | | 38 | noO | H30 | CTX-M-15, TEM-1 | SRR8360101 |
| 61 | | M043A | D | | 38 | noO | H18 | CTX-M-15 | ~ |
| 62 | | M030X | D | | 115 | O92 | H31 | CTX-M-14, TEM-1 | SRR8535171 |
| 63 | | B099X | D | | 115 | O92 | H31 | CTX-M-15 | SRR8984098 |
| 64 | | M033A | D | | 449 | noO | H18 | CTX-M-14, TEM-1 | SRR8360098 |
| 65 | | B011X | D | | 449 | O15 | H18 | CTX-M-15, TEM-1, OXA-1 | SRR8360103 |
| 66 | | M011A | D | | 449* | noO | noH | CTX-M-14 | SRR8535138 |
| 67 | | B011A | D | | 449 | O15 | H18 | CTX-M-15, TEM-1, OXA-1 | SRR8360252 |
|  | | | * 6/7 alleles as per the sequence type, 7th allele could not be positively identified. | | | | | | |
|  | | | † O:H serotype as predicted using the Serotypefinder database. | | | | | | |
|  | | | ‡ order in tree shown in Figure 2, from top to bottom. | | | | | | |
|  | | | ~ uploaded to SRA, awaiting accession number | | | | | | |

**Table S6***.* Mothers’ isolates categorized by phylogroup and Multilocus Sequence Typing (MLST). Frequency (%) of isolates in each category is shown.

|  |  | Phylogroup | | | | |
| --- | --- | --- | --- | --- | --- | --- |
|  |  | **A** | **B1** | **B2** | **D** | **F** |
| MLST | **38** | 0 (0) | 0 (0) | 0 (0) | 4 (50) | 0 (0) |
|  | **95** | 0 (0) | 0 (0) | 2 (12.5) | 0 (0) | 0 (0) |
|  | **115** | 0 (0) | 0 (0) | 0 (0) | 1 (12.5) | 0 (0) |
|  | **127** | 0 (0) | 0 (0) | 2 (12.5) | 0 (0) | 0 (0) |
|  | **131** | 0 (0) | 0 (0) | 9 (56.3) | 0 (0) | 0 (0) |
|  | **165** | 1 (12.5) | 0 (0) | 0 (0) | 0 (0) | 0 (0) |
|  | **202** | 1 (12.5) | 0 (0) | 0 (0) | 0 (0) | 0 (0) |
|  | **224** | 0 (0) | 1 (33.3) | 0 (0) | 0 (0) | 0 (0) |
|  | **226** | 2 (25.0) | 0 (0) | 0 (0) | 0 (0) | 0 (0) |
|  | **443** | 0 (0) | 0 (0) | 0 (0) | 0 (0) | 0 (0) |
|  | **449** | 0 (0) | 0 (0) | 0 (0) | 1 (12.5) | 0 (0) |
|  | **617** | 1 (12.5) | 0 (0) | 0 (0) | 0 (0) | 0 (0) |
|  | **636** | 0 (0) | 0 (0) | 1 (6.25) | 0 (0) | 0 (0) |
|  | **648** | 0 (0) | 0 (0) | 0 (0) | 0 (0) | 6 (50.0) |
|  | **1193** | 0 (0) | 0 (0) | 1 (6.3) | 0 (0) | 0 (0) |
|  | **1485** | 0 (0) | 0 (0) | 0 (0) | 0 (0) | 4 (33.3) |
|  | **1722** | 0 (0) | 0 (0) | 0 (0) | 0 (0) | 2 (16.7) |
|  | **1730** | 0 (0) | 1 (33.3) | 0 (0) | 0 (0) | 0 (0) |
|  | **2161** | 0 (0) | 1 (33.3) | 0 (0) | 0 (0) | 0 (0) |
|  | **4981** | 1 (12.5) | 0 (0) | 0 (0) | 0 (0) | 0 (0) |
|  | **6900** | 1 (12.5) | 0 (0) | 0 (0) | 0 (0) | 0 (0) |
|  | **unknown** | 1 (12.5) | 0 (0) | 1 (12.5) | 2 (12.5) | 0 (0) |
|  | **Total** | 8 (17.0) | 3 (6.4) | 16 (34.0) | 8 (17.0) | 12 (25.5) |

**Table S7**. Infants’ isolates categorized by phylogroup and Multilocus Sequence Typing (MLST). Frequency (%) of isolates in each category are shown.

|  |  | Phylogroup | | | | |
| --- | --- | --- | --- | --- | --- | --- |
|  |  | **A** | **B1** | **B2** | **D** | **F** |
| MLST | **38** | 0 (0) | 0 (0) | 0 (0) | 3 (50) | 0 (0) |
|  | **95** | 0 (0) | 0 (0) | 1 (10) | 0 (0) | 0 (0) |
|  | **115** | 0 (0) | 0 (0) | 0 (0) | 1 (16.7) | 0 (0) |
|  | **127** | 0 (0) | 0 (0) | 0 (0) | 0 (0) | 0 (0) |
|  | **131** | 0 (0) | 0 (0) | 8 (80) | 0 (0) | 0 (0) |
|  | **165** | 0 (0) | 0 (0) | 0 (0) | 0 (0) | 0 (0) |
|  | **202** | 0 (0) | 0 (0) | 0 (0) | 0 (0) | 0 (0) |
|  | **224** | 0 (0) | 0 (0) | 0 (0) | 0 (0) | 0 (0) |
|  | **226** | 0 (0) | 0 (0) | 0 (0) | 0 (0) | 0 (0) |
|  | **443** | 0 (0) | 1 (100) | 0 (0) | 0 (0) | 0 (0) |
|  | **449** | 0 (0) | 0 (0) | 0 (0) | 2 (33.3) | 0 (0) |
|  | **617** | 1 (100) | 0 (0) | 0 (0) | 0 (0) | 0 (0) |
|  | **636** | 0 (0) | 0 (0) | 1 (10) | 0 (0) | 0 (0) |
|  | **648** | 0 (0) | 0 (0) | 0 (0) | 0 (0) | 1 (50) |
|  | **1193** | 0 (0) | 0 (0) | 0 (0) | 0 (0) | 0 (0) |
|  | **1485** | 0 (0) | 0 (0) | 0 (0) | 0 (0) | 0 (0) |
|  | **1722** | 0 (0) | 0 (0) | 0 (0) | 0 (0) | 1 (50) |
|  | **1730** | 0 (0) | 0 (0) | 0 (0) | 0 (0) | 0 (0) |
|  | **2161** | 0 (0) | 0 (0) | 0 (0) | 0 (0) | 0 (0) |
|  | **4981** | 0 (0) | 0 (0) | 0 (0) | 0 (0) | 0 (0) |
|  | **6900** | 0 (0) | 0 (0) | 0 (0) | 0 (0) | 0 (0) |
|  | **Total** | 1 (5) | 1 (5) | 10 (50) | 6 (30) | 2 (10) |

1. Shaw KJ, Rather PN, Hare RS, Miller GH. Molecular genetics of aminoglycoside resistance genes and familial relationships of the aminoglycoside-modifying enzymes. Microbiol Rev. 1993;57:138–63.

2. Frasson I, Cavallaro A, Bergo C, Richter SN, Palù G. Prevalence of aac(6′)-Ib-cr plasmid-mediated and chromosome-encoded fluoroquinolone resistance in Enterobacteriaceae in Italy. Gut Pathog. 2011;3.

3. Bush K, Jacoby GA. Updated Functional Classification of-Lactamases. Antimicrob Agents Chemother [Internet]. 2010;54:969–76. Available from: http://www.lahey.org

4. Schwarz S, Kehrenberg C, Doublet B, Cloeckaert A. Molecular basis of bacterial resistance to chloramphenicol and florfenicol. FEMS Microbiol Rev. 2004;28:519–42.

5. Brolund A, Sundqvist M, Kahlmeter G, Grape M. Molecular characterisation of trimethoprim resistance in Escherichia coli and Klebsiella pneumoniae during a two year intervention on trimethoprim use. PLoS One. 2010;5.

6. Nguyen MCP, Woerther PL, Bouvet M, Andremont A, Leclercq R, Canu A. Escherichia coli as reservoir for macrolide resistance genes. Emerg Infect Dis. 2009;15:1648–50.

7. Ito R, Pacey MP, Mettus RT, Sluis-Cremer N, Doi Y. Origin of the plasmid-mediated fosfomycin resistance gene fosA3. J Antimicrob Chemother. Oxford University Press; 2018;73:373–6.

8. Takahata S, Ida T, Hiraishi T, Sakakibara S, Maebashi K, Terada S, et al. Molecular mechanisms of fosfomycin resistance in clinical isolates of Escherichia coli. Int J Antimicrob Agents. Elsevier; 2010;35:333–7.

9. Liu YY, Wang Y, Walsh TR, Yi LX, Zhang R, Spencer J, et al. Emergence of plasmid-mediated colistin resistance mechanism MCR-1 in animals and human beings in China: A microbiological and molecular biological study. Lancet Infect Dis. Lancet Publishing Group; 2016;16:161–8.

10. Edgar R, Bibi E. MdfA, an Escherichia coli multidrug resistance protein with an extraordinarily broad spectrum of drug recognition. J Bacteriol [Internet]. 1997 [cited 2019 Aug 20];179:2274–80. Available from: http://www.ncbi.nlm.nih.gov/pubmed/9079913

11. Kücken D, Feucht HH, Kaulfers PM. Association of qacE and qacEΔ1 with multiple resistance to antibiotics and antiseptics in clinical isolates of Gram-negative bacteria. FEMS Microbiol Lett [Internet]. 2000 [cited 2019 Aug 20];183:95–8. Available from: https://academic.oup.com/femsle/article-lookup/doi/10.1111/j.1574-6968.2000.tb08939.x

12. Kazama H, Hamashima H, Sasatsu M, Arai T. Distribution of the antiseptic-resistance genes qacE and qacE delta 1 in Gram-negative bacteria. FEMS Microbiol Lett [Internet]. Oxford University Press (OUP); 1998 [cited 2020 Dec 7];159:173–8. Available from: https://pubmed.ncbi.nlm.nih.gov/9503610/

13. Sorlozano A, Gutierrez J, Jimenez A, Luna JDD, Martínez JL. Contribution of a new mutation in parE to quinolone resistance in extended-spectrum-β-lactamase-producing Escherichia coli isolates. J Clin Microbiol. 2007;45:2740–2.

14. Salah FD, Soubeiga ST, Ouattara AK, Sadji AY, Metuor-Dabire A, Obiri-Yeboah D, et al. Distribution of quinolone resistance gene (qnr) in ESBL-producing Escherichia coli and Klebsiella spp. in Lomé, Togo. Antimicrob Resist Infect Control [Internet]. BioMed Central Ltd.; 2019 [cited 2020 Dec 8];8:104. Available from: https://aricjournal.biomedcentral.com/articles/10.1186/s13756-019-0552-0

15. Sköld O. Sulfonamide resistance: Mechanisms and trends. Drug Resist Updat. Churchill Livingstone; 2000;3:155–60.

16. Roberts MC. Update on acquired tetracycline resistance genes. FEMS Microbiol Lett. Elsevier; 2005;245:195–203.
